# Identification of ncRNA Biomarkers in Non–Small Cell Lung Cancer to Address Racial Disparities

**DOI:** 10.1158/2767-9764.CRC-24-0262

**Published:** 2024-12-27

**Authors:** Lu Gao, Pushpa Dhilipkannah, Feng Jiang

**Affiliations:** Department of Pathology, University of Maryland School of Medicine, Baltimore, Maryland.; Department of Pathology, University of Maryland School of Medicine, Baltimore, Maryland.; Department of Pathology, University of Maryland School of Medicine, Baltimore, Maryland.

## Abstract

Lung cancer significantly impacts mortality, with African Americans (AA) showing higher rates than White Americans (WA). ncRNAs play a crucial role in lung tumorigenesis. To identify ncRNA biomarkers associated with racial disparities in lung cancer, we used droplet digital PCR to examine 93 lung cancer–associated ncRNAs in plasma and sputum samples from participants of AA and WA backgrounds, including 118 patients and 92 cancer-free smokers. In the AA population, plasma showed differential expression of 10 ncRNAs, whereas sputum revealed four ncRNAs when comparing patients with lung cancer to the control group. In the WA population, the plasma displayed 11 ncRNAs, and the sputum had five ncRNAs showing differential expression between patients with lung cancer and the control group. For AAs, we identified a three-ncRNA panel (plasma miRs–147b, 324-3p, and 422a) for diagnosing lung cancer in AAs with 86% sensitivity and 89% specificity. For WAs, a four-ncRNA panel comprising sputum miR-34a-5p and plasma miRs–103-3p, 126-3p, and 205-5p was developed, achieving 88% sensitivity and 87% specificity. These panels remained effective across various stages and types of lung tumors and were validated in an independent cohort of 56 patients and 72 controls. Ethnicity-related ncRNA signatures hold promise as biomarkers for tackling racial disparities in patients with lung cancer.

**Significance:**

This study identifies ethnicity-related ncRNA biomarkers that differentiate lung cancer in AAs and WAs, offering diagnostic panels with high sensitivity and specificity. These findings provide a promising approach to addressing racial disparities in lung cancer detection and improving early diagnosis across diverse populations.

## Introduction

Lung cancer is the leading cause of cancer-related deaths in both men and women in the United States (1). Non–small cell lung cancer (NSCLC) accounts for 85% of all lung cancer cases and is mainly composed of two histologic types: adenocarcinoma and squamous cell carcinoma (1). Early detection and timely treatment can significantly reduce morbidity and mortality in NSCLC. However, the existing diagnostic methods fall short in the early detection of NSCLC. Furthermore, there are notable disparities in NSCLC between different ethnicities, with African Americans (AA) experiencing a higher prevalence and mortality rate (2). The annual incidence of lung cancer is the highest among AAs at 76.1 per 100,000, followed by White Americans (WA) at 69.7/100,000, American Indians/Alaska Natives at 48.4/100,000, and Asian/Pacific Islanders at 38.4 per 100,000 (2). In addition to socioeconomic differences, biological factors such as tumor biology, genetics, and molecular alterations also contribute to disparities in lung cancer. For instance, genome-wide association studies have identified lung cancer susceptibility loci on chromosomes 5p15 and 15q25 in AA populations (3). Differences in the methylation levels of genes with functional relevance, such as nuclear receptor subfamily 3, have been identified as potential contributors to racial disparities in NSCLC (4). EGFR mutations are more common in AA patients with lung cancer than in other populations (5). mRNA transcripts from AAs are less likely to undergo alternative polyadenylation in lung cancer than those from WAs (6). Furthermore, elevated levels of cytokines, including IL-1β, IL-10, and TNF-α, are associated with an increased risk of lung cancer in AA patients (7, 8). The molecular and genetic variations linked to lung cancer disparities offer potential as biomarkers for NSCLC in AAs, which could address the observed ethnic disparities in lung cancer treatment and outcomes.

ncRNAs are RNA molecules that are not translated into proteins but are essential in regulating gene expression and cellular processes. ncRNAs mainly consist of miRNAs, long noncoding RNAs (lncRNA), and small nucleolar RNAs (snoRNA), among others. Aberrant expression of certain ncRNAs has been closely linked to the onset and progression of cancer, highlighting their potential as therapeutic targets and diagnostic markers. Furthermore, miRNAs are implicated in disparities observed between AAs and other populations (9). Numerous studies, including ours, have identified miRNAs, lncRNAs, and snoRNAs linked to lung cancer, underscoring their potential as biomarkers for this disease (9). We postulate that by studying and distinguishing ncRNA patterns in the plasma and sputum of patients with NSCLC from both the AA and WA cohorts, we can develop noninvasive lung cancer biomarkers tailored to individual ethnicities.

## Materials and Methods

### Patients and research design

Eligible participants were current or former smokers ages 50 to 80 years. We excluded those who were pregnant or lactating, those with current pulmonary infections, individuals who had undergone thoracic surgery within the last 6 months, those who had received chest radiotherapy in the preceding year, and those with a life expectancy of <1 year. Demographic and clinical data, including age, sex, race, and smoking history, were collected from medical records. To confirm malignancy, tissue samples obtained either surgically or via biopsy were subjected to a pathologic examination. Surgical pathologic staging adhered to the tumor–node–metastasis classification of the International Union Against Cancer, the American Joint Committee on Cancer, and the International Staging System for Lung Cancer. Histopathologic classification was based on guidelines provided by the World Health Organization. Radiographic characteristics of pulmonary nodules (PN) were derived from CT images. These included the maximum transverse size, visually determined type (categorized as nonsolid, ground-glass opacity, part-solid, solid, peri fissure, or spiculation), and lung location of the nodule. A benign diagnosis was confirmed either pathologically, specifying a benign cause, or through the clinical and radiographic stability of the PNs observed during multiple check-ups over a 2-year follow-up period. A total of 340 participants were enrolled in this study. Among the 340 participants, 174 patients were diagnosed with NSCLC, with an equal distribution of 87 AA and 87 WA patients with lung cancer. The other 166 patients had benign conditions: 99 had granulomatous inflammation, 38 exhibited nonspecific inflammatory changes, and 29 presented with lung infections. The cohort was bifurcated randomly into training and validation sets, as detailed in Tables 1 and 2.

**Table 1:** Characteristics of a training set of patients with NSCLC and cancer-free smokers

	NSCLC cases (*n* = 118)	Controls (*n* = 92)
Age	67.25 (SD 12.33)	66.29 (SD 11.19)
Sex		
Female	46	36
Male	72	56
Race		
AAs	59	46
WAs	59	46
Smoking pack-years (median)	31.6	30.9
PN size (mm)	19.37 (SD 12.16)	6.12 (SD 4.58)
Stage		
Stage I	78	
Stage II	26	
Stages III–IV	14	
Histologic type		
Adenocarcinoma	69	
Squamous cell carcinoma	49	

**Table 2:** Characteristics of a validation set of patients with NSCLC and cancer-free smokers

	NSCLC cases (*n* = 56)	Controls (*n* = 72)
Age	67.84 (SD 11.73)	67.91 (SD 11.07)
Sex		
Female	20	25
Male	36	47
Race		
AAs	28	36
WAs	28	36
Smoking pack-years (median)	35.1	34.6
PN size (mm)	20.27 (SD = 11.46)	7.48 (SD = 5.13)
Stage		
Stage I	26	
Stage II	24	
Stages III–IV	6	
Histologic type		
Adenocarcinoma	32	
Squamous cell carcinoma	24	

### Sputum and blood sample collection and preparation

Specimens were obtained from participants prior to treatment initiation. For sputum collection, the participants were instructed to blow their nose, rinse their mouths, and drink water to minimize contamination from oral epithelial cells. The sputum was then collected in sterile containers and promptly processed on ice using 0.1% dithiothreitol and PBS (Sigma-Aldrich; refs. 10, 11). Concurrently, blood samples were drawn, and within an hour of collection, plasma was separated following standard clinical protocols (10, 11).

### RNA isolation

RNA was extracted from the specimens using the miRNeasy Mini Kit spin column (QIAGEN), as previously described (10, 11). The extracted RNA samples were promptly stored at −80°C in barcoded cryotubes.

### Droplet digital PCR analysis of miRNAs, lncRNAs, and snoRNAs

Numerous studies, including our own, have identified 93 specific miRNAs, lncRNAs, and snoRNAs in tissue specimens related to lung cancer, suggesting their potential as valuable biomarkers for the disease (Supplementary Table S1). In this study, we employed ddPCR analysis for 93 ncRNAs in both plasma and sputum, following previously developed methods (10, 11). One microliter of RNA from each sample was reverse transcribed using gene-specific primers for each target using the TaqMan miRNA RT Kit (Applied Biosystems). For the droplet digital PCR (ddPCR) reactions, a mixture containing 5 μL cDNA solution, 10 μL Supermix, and 1 μL TaqMan primer/probe mix was prepared in a 20 μL volume. This mixture was loaded into cartridges filled with droplet generation oil (Bio-Rad) and placed in a QX100 Droplet Generator (Bio-Rad). The droplets formed were transferred to a 96-well PCR plate, followed by PCR amplification using a T100 thermal cycler (Bio-Rad). The ddPCR method generated more than 10,000 droplets per well, which were subsequently analyzed using a fluorescence detector. This ensured that ncRNAs were consistently and accurately detected in the clinical samples. We assessed the number of positive reactions and employed Poisson’s distribution to accurately determine the concentration of the target genes (10, 11).

To ensure that the ncRNA expression levels are reliable and reproducible across different samples, we included a nonhuman miRNA, cel-miR-39, as an exogenous control in both plasma and sputum samples. A synthetic cel-miR-39 RNA oligonucleotide (Integrated DNA Technologies, Inc.) was prepared at a concentration of 25 fmol in 5 μL of nuclease-free water and added to each plasma or sputum sample after the addition of a 2× denaturing solution to prevent degradation by endogenous RNases. Because ddPCR results are represented as copy numbers per microliter (copies/μL) instead of cycle threshold values, we normalized miRNA expression to cel-miR-39 in each sample using the following method: First, we calculated a normalization factor for each sample using the copy number of cel-miR-39. This factor represents the efficiency and consistency of the RNA extraction and reverse transcription processes: Normalization factor = (copy number of cel-miR-39 in sample)/(average copy number of cel-miR-39 across all samples). To normalize the target ncRNA, we adjusted the copy number of the target miRNA in each sample by dividing it by the normalization factor calculated in the previous step: Normalized miRNA copy number = (copy number of target mirna)/(normalization factor). This approach ensured that the normalized ncRNA expression levels were consistent and accurate across different samples, accounting for any technical variations in the RNA extraction and reverse transcription processes.

### Statistical analysis

Statistical significance for biomarkers and clinical determinants was ascertained using the Mann–Whitney *U* test or *χ*^2^ test. Pearson’s correlation was used to assess the relationship between miRNA expression and clinical and demographic data, including smoking history measured in pack-years. The construction of a lung cancer biomarker panel was strategized by bifurcating the cohort into training and validation subsets and adhering to the guidelines proposed by the NCI’s Early Detection Research Network. In the training subset, feature selection was conducted using the least absolute shrinkage and selection operator in tandem with logistic regression. A 10-fold cross-validation, reinforced with bootstrapping, was used to mitigate the influence of outliers. The mean decrease in Gini impurity served as the metric for evaluating variable importance, with the FDR addressing multiple testing corrections. Discrimination metrics were established using ROC curve analysis, reporting the AUC values accompanied by 95% confidence intervals. The confidence intervals for the performance metrics (AUC, sensitivity, and specificity) were determined by employing an assortment of statistical methodologies. After refining the diagnostic panels from the training set, we assessed their robustness in the validation subset using the AUC, sensitivity, and specificity.

### Data availability

The data generated in this study are available upon request from the corresponding author.

## Results

### Differential expression of ncRNAs in plasma and sputum of patients with NSCLC versus cancer-free smokers

The expression levels of 93 lung cancer–associated ncRNAs, which included 67 miRNAs, 21 snoRNAs, and five lncRNAs (Supplementary Table S1), were quantified in plasma and sputum using a microplate-based ddPCR technique (10, 11) This analysis was conducted on specimens obtained from 59 AA patients with NSCLC, 46 cancer-free AA smokers, 59 WA patients with NSCLC, and 46 cancer-free WA smokers (Table 1). In plasma, differential expression of 25 ncRNAs, including 18 miRNAs, five snoRNAs and two lncRNAs, was observed between patients with cancer and cancer-free smokers across all ethnicities (Mann–Whitney *U* test: *P* < 0.05; FDR-adjusted *P* < 0.05; Supplementary Table S2). Similarly, in sputum, eight ncRNAs, comprising three miRNAs, three snoRNAs, and two lncRNAs, exhibited differential expression between patients with NSCLC and cancer-free smokers (Mann–Whitney *U* test: *P* < 0.05; FDR-adjusted *P* < 0.05; Supplementary Table S2).

### Differential expression of ncRNAs in plasma and sputum among individuals from different ethnic populations

**Figure 1:**
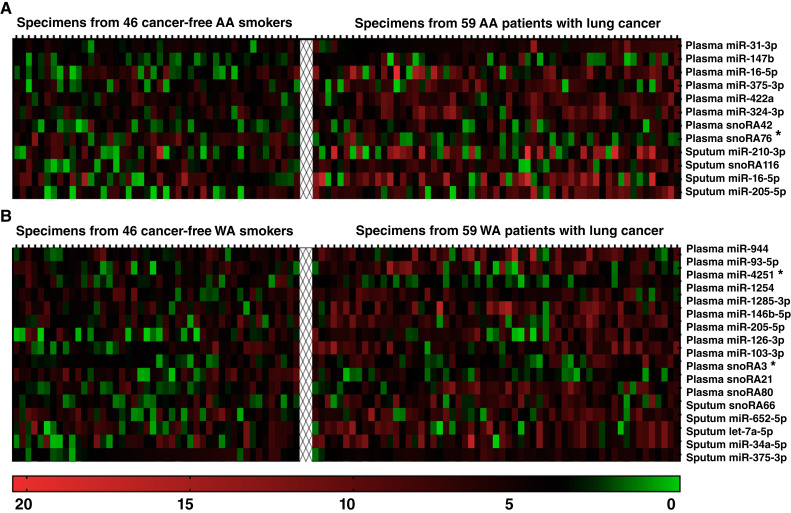
Differential expression heatmap of ncRNAs in plasma and sputum samples from AA and WA patients with lung cancer vs. their healthy counterparts. **A,** Among 59 AA patients with lung cancer and 46 cancer-free AA smokers, 12 ncRNAs showed statistically significant expression differences (all *P* < 0.05). **B,** In a comparison between 59 WA patients with lung cancer and 46 cancer-free WA smokers, 17 genes revealed statistically significant expression differences with a *P* value < 0.05. The color scale spans from green (indicating downregulation) to black (no change) and red (signifying upregulation). *ncRNAs with statistically significant reductions in expression in specimens from patients with lung cancer compared with specimens from cancer-free smokers.

We further explored the differential expression of ncRNAs in plasma and sputum across individual ethnic groups. Plasma samples from AA participants showed that seven ncRNAs (miRs-31-5p, 147b, 16-5p, 375-3p, 422a, 324-3p, and snoRA42) showed a significant increase in expression in patients with lung cancer compared with cancer-free AA controls (Mann–Whitney *U* test: *P* < 0.05; FDR-adjusted *P* < 0.05; Table 3; Fig. 1; Supplementary Fig. S1). However, snoRA76 displayed a significant decrease in expression in AA patients with lung cancer compared with that in controls (Mann–Whitney *U* test: *P* < 0.05; FDR-adjusted *P* < 0.05; Table 3; Fig. 1; Supplementary Fig. S1). In the sputum samples from AA participants, four ncRNAs, three microRNAs (miRs-16-5p, 210-3p, and 205-5p), and one snoRNA (snoRA116) were found to be elevated in patients with lung cancer as compared with cancer-free AA controls (Mann–Whitney *U* test: *P* < 0.05; FDR-adjusted *P* < 0.05; Table 3; Fig. 1A; Supplementary Fig. S1).

**Table 3:** Set of 12 ncRNAs found to be significantly differential expressed in AAs, as analyzed by the Mann–Whitney *U* test, with a FDR-adjusted *P* value of less than 0.05

ncRNA	Mean expression (patients with cancer)	Mean expression (controls)	Fold change	Mann–Whitney *U* statistic	FDR-adjusted *P* value
Plasma miR-31-5p	2.115	1.813	1.167	864	0.0005
Plasma miR-147b	1.7	1.31	1.298	875	0.0016
Plasma miR-16-5p	2.793	1.789	1.561	953	0.0086
Plasma miR-375-3p	2.413	1.76	1.371	870	0.0015
Plasma miR-422a	2.376	1.964	1.21	827	0.0005
Plasma miR-324-3p	2.47	2.01	1.229	1232	0.0126
Plasma snoRA42	1.783	1.57	1.136	1336	0.0407
Plasma snoRA76	1.391	2.199	0.633	1600	0.0205^a^
Sputum miR-210-3p	2.461	1.678	1.467	1485	0.0041
Sputum snoRA116	2.315	1.788	1.295	1480	0.0038
Sputum miR-16-5p	2.793	1.766	1.582	1374	0.0007
Sputum miR-205-5p	2.647	1.984	1.334	1284	0.0001

a The ncRNA was significantly decreased in expression in AA patients with lung cancer compared with cancer-free AA smokers.

In plasma samples from WA participants, 12 ncRNAs were differentially expressed between patients with lung cancer and cancer-free WA smokers. These ncRNAs included eight microRNAs (miRs-93-5p, 103a-3p, 126-3p, 146b-5p, 205-5p, 944, 4251, and 1285-3p) and three snoRNAs (snoRA3, snoRA21, and snoRA80; Mann–Whitney *U* test: *P* < 0.05; FDR-adjusted *P* < 0.05; Table 4; Fig. 1B; Supplementary Fig. S2). Ten of these ncRNAs (miRs-93-5p, 103a-3p, 126-3p, 146b-5p, 205-5p, 944, 1254, and 1285-3p, and snoRA21 and snoRA80) showed increased expression, whereas two ncRNAs (miR-4251 and snoRA3) had decreased expression in WA patients with lung cancer when compared with cancer-free AA smokers. In sputum samples from WA patients, five ncRNAs demonstrated elevated expression in patients with lung cancer compared with cancer-free WA smokers. These ncRNAs included four miRNAs: miR-34a-5p, miR-652-5p, miR-375-3p, and let-7a, along with one snoRNA: snoRA66 (Mann–Whitney *U* test: *P* < 0.05; FDR-adjusted *P* < 0.05; Table 4; Fig. 1; Supplementary Fig. S2).

**Table 4:** Set of 17 ncRNAs found to be significantly differential expressed in WAs, as analyzed by the Mann–Whitney *U* test, with a FDR-adjusted *P* value of less than 0.05

ncRNA	Mean expression (patients with cancer)	Mean expression (controls)	Fold change	Mann–Whitney *U* statistic	FDR-adjusted *P* value
Plasma miR-944	2.279	1.738	1.311	897	0.0027
Plasma miR-93-5p	2.325	1.531	1.519	1007	0.0123
Plasma miR-4251	1.328	1.826	0.727	1003	0.0115^a^
Plasma miR-1254	2.162	1.595	1.355	1032	0.0262
Plasma miR-1285-3p	2.531	1.947	1.3	884	0.002
Plasma miR-146b-5p	2.529	1.628	1.553	975	0.0132
Plasma miR-205-5p	2.228	1.489	1.436	911	0.0037
Plasma miR-126-3p	2.333	1.714	1.361	773	0.0001
Plasma miR-103-3p	1.681	1.315	1.278	909	0.0051
Plasma snoRA3	1.195	1.473	0.811	932	0.0057^a^
Plasma snoRA21	2.317	1.618	1.432	875	0.0017
Plasma snoRA80	2.226	1.546	1.44	846	0.0008
Sputum snoRA66	2.385	1.881	1.268	1038	0.0213
Sputum miR-652-5p	2.463	1.984	1.241	1027	0.0325
Sputum let-7a-5p	2.376	1.555	1.528	907	0.0034
Sputum miR-34a-5p	1.998	1.541	1.297	1024	0.0317
Sputum miR-375-3p	2.137	1.844	1.159	984	0.0157

a The ncRNA was significantly decreased in expression in AA patients with lung cancer compared with cancer-free AA smokers.

### The diagnostic utility of the plasma and sputum ncRNA biomarkers varies with ethnicity

**Figure 2:**
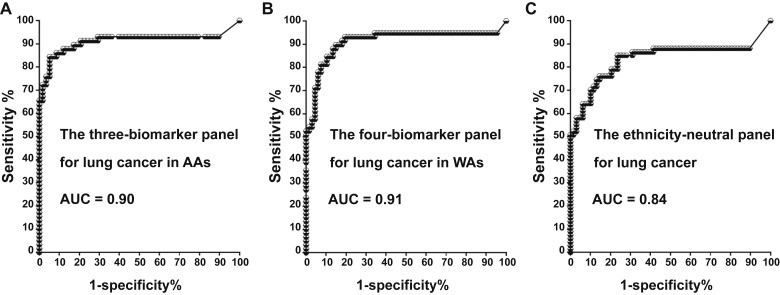
Performance of the three individual panels for diagnosing lung cancer in different ethnic groups in the training set. **A,** ROC curves depict the accuracy of the three-biomarker panel in diagnosing lung cancer in AAs, yielding an AUC of 0.90. **B,** ROC curves represent the accuracy of the four-biomarker panel in diagnosing lung cancer in WAs, with an AUC of 0.91. **C,** The pan-ethnic biomarker panel displays an AUC of 0.84 in the diagnosis of lung cancer, irrespective of racial populations.

We used logistic regression and a backward elimination approach to identify specific ncRNA biomarker panels for lung cancer in different ethnicities. For AAs, the best prediction for lung cancer was achieved using a combination of three ncRNAs: miRs-147b, 324-3p, and 422a in plasma. This panel yielded an AUC of 0.90, distinguishing AA patients with cancer from healthy AAs with a sensitivity of 86% and specificity of 89% (Fig. 2; Table 5). For WAs, optimal prediction was derived from a combination of four ncRNAs: sputum miR-34a-5p, plasma miR-103-3p, plasma 126-3p, and plasma 205-5p. This combination achieved an AUC of 0.91, diagnosing NSCLC with a sensitivity of 89% and specificity of 87% (all *P* < 0.05; Table 5). Additionally, for pan-ethnic diagnosis, a panel comprising plasma miR-21-3p, plasma miR-210-3p, and sputum miR-126-3p demonstrated the best universal diagnostic ability. This combination achieved an AUC of 0.84, with a sensitivity of 71% and specificity of 88% (Fig. 2; Table 5). The pan-ethnic biomarker panel exhibited lower sensitivity for AAs and WAs than their individual biomarker panels (71% vs. 86% for AAs and 89% for WAs, *P* < 0.05), while maintaining similar specificity (Table 5). Among the 10 ncRNA biomarkers, plasma miR-205-5p and sputum miR-126-3p were associated with age, whereas plasma miR-422a and plasma miR-324-3p were related to the sex of the patients (all *P* values <0.05; Supplementary Table S3). Plasma miR-422a was associated with the size of PNs, and plasma miR-147b levels were correlated with tumor stage. ncRNAs were not associated with smoking history (Supplementary Table S3). When these biomarkers were used in combination as panels, their diagnostic values did not show any association with the patients’ age, sex, smoking history, PN size, tumor stage, or histologic type of lung tumor.

**Table 5:** Diagnostic values of the three individual panels in the training set

Three individual panels	Sensitivity, % (95% CI)	Specificity, % (95% CI)
The three-biomarker panel in AAs	86.44% (75.02%–93.96%)	89.13% (76.43%–96.38%)
The four-biomarker panel in WAs	89.83% (79.17%–96.18%)	86.96% (73.74%–95.06%)
The pan-ethnic biomarker panel	71.19% (62.13%–79.15%)	88.04% (79.61%–93.88%)

Abbreviation: CI, confidence interval.

### Verifying the diagnostic potential of the biomarker panels for disparities

We validated three distinct ncRNA biomarker panels for lung cancer diagnosis in the validation cohort. For AAs, the biomarker panel comprising plasma miR-147b, miR-324-3p, and miR-422a achieved a sensitivity of 86% and specificity of 89% in detecting lung cancer (Supplementary Table S4). For WAs, the panel including sputum miR-34a-5p, plasma miR-103-3p, plasma 126-3p, and plasma 205-5p demonstrated a sensitivity of 89% and specificity of 86% (Supplementary Table S4). The ethnicity-neutral biomarker panel, including plasma miR-21-3p, plasma miR-210-3p, and sputum miR-126-3p, achieved a sensitivity of 71% and specificity of 89% in the diagnosis of lung cancer across all ethnic groups (Supplementary Table S4). The results in the validation set confirm the findings in the training set and thus support the potential of these biomarkers for early NSCLC detection in different racial populations.

## Discussion

The National Lung Screening Trial has established that low-dose CT (LDCT) screening significantly reduces lung cancer–related mortality among high-risk populations, notably smokers (1). Currently, LDCT is used for lung cancer screening in smokers. However, this method has significantly increased the detection of indeterminate PNs in asymptomatic individuals. Of the smokers screened, 24.2% were found to have indeterminate PNs on LDCT, and 96.4% of these nodules were subsequently confirmed as benign growths (1). Moreover, although CT screening using LDCT boasts a sensitivity exceeding 90%, its specificity is only 61% (1), resulting in a substantial false-positive rate or overdiagnosis (1). Given the notably high incidence and mortality rates among AAs, there is an urgent need for noninvasive molecular biomarkers tailored to AA demographics. These biomarkers can facilitate the early detection of NSCLC either when used independently or in conjunction with LDCT, aiming to reduce the false-positive rates frequently associated with LDCT. Although previous investigations have revealed certain miRNA variations in surgically resected lung tumor tissues between AA and WA patients (12, 13), the field still lacks noninvasive molecular biomarkers tailored for early lung cancer detection in AA patients.

In this study, we systematically analyzed 93 lung cancer–related ncRNAs from plasma and sputum samples of both AA and WA patients with lung cancer as well as from cancer-free controls. Distinct ncRNA alterations associated with each population were identified, leading to the development of specific diagnostic panels for each group. Moreover, we developed an ethnicity-neutral biomarker panel for lung cancer diagnosis. However, this pan-ethnic biomarker panel demonstrated suboptimal diagnostic sensitivity among various ethnic groups compared with population-specific markers. Furthermore, although some ncRNAs are associated with age, sex, size of PNs, and smoking history, the combined use of these genes as biomarker panels was not influenced by these factors in either population. Interestingly, their diagnostic efficacy remains consistent across the early and late stages of lung tumors, underscoring their potential for early NSCLC detection in clinical contexts. Additionally, these biomarkers were not associated with the PNs identified via LDCT. Thus, these biomarkers may be instrumental in distinguishing lung cancer within PNs identified by LDCT, potentially reducing the elevated false-positive rate. Nonetheless, a more extensive study with a broader cohort is required to validate this diagnostic potential.

Among AAs, miRs-147b, 324-3p, and 422a are the most discriminatory biomarkers for the diagnosis of lung cancer. miR-147b can promote lymph node metastasis and cancer prognosis by regulating PRPF4B, WDR82, and NR3C2 (14). In addition, it influences drug resistance to EGFR inhibitors by modulating the tricarboxylic acid cycle. miR-324-3p is highly expressed in lung cancer cells and promotes their growth and invasion (15). miR-422a can inhibit the TGF-β/SMAD pathway by downregulating sulfatase 2 and hence constrain NSCLC cell proliferation, migration, invasion, colony formation, epithelial–mesenchymal transition (EMT), and tumorigenesis (16). Among WAs, miR-34a-5p, miR-103-3p, miR-126-3p, and 205-5p are important for the diagnosis of lung cancer. miR-34-5p is implicated in the regulation of tumor growth due to its role in EMT via EMT transcription factors, p53, and other important signaling pathways (17). Dysfunction of miR-103-3p is pivotal in lung tumorigenesis, as it directly targets PDCD10, influencing lung cancer cell proliferation and metastasis (18). The miR-103/PDCD10 signaling pathway is a potential novel therapeutic target for NSCLC treatment (18). miR-126-3p, an endothelial miRNA, is aberrantly expressed in specimens from patients with lung cancer (19). Its reintroduction curbs tumor growth by targeting EGFL7. Elevated expression of miR-205-5p has been implicated in the initiation and progression of NSCLC (20). This miRNA is also associated with EMT modulation by targeting EMT-related genes, which in turn affects the invasive and metastatic capabilities of lung cancer cells (21). Furthermore, miR-205-5p is believed to contribute to carcinogenesis and chemoresistance of NSCLC by influencing the PTEN signaling pathway (22). Nevertheless, further investigations are needed to fully understand the specific roles and implications of these ncRNAs in accounting for racial disparities in lung cancer incidence.

This study provides valuable insights but also highlights areas requiring further exploration. Expanding the sample size could uncover less discriminatory markers. Although this study focused on analyzing 93 ncRNAs, numerous other genes await systematic validation in future research. In our ongoing study, we are using a combined score of these biomarkers to enhance sensitivity and specificity compared with individual cutoff values. By applying logistic regression models and backward elimination, we aim to derive optimal ncRNA panels for both AAs and WAs, achieving high diagnostic accuracy. Additionally, a longitudinal study is warranted to investigate how these molecules relate to disease pathology and progression over time among populations of different races. We also observed distinct ncRNA expression patterns between plasma and sputum from cancer cases and controls. Some ncRNAs differed in plasma but not in sputum and *vice versa*, whereas others showed similar patterns in both. This can be due to tissue-specific expression, different release mechanisms, and varying stability between plasma and sputum. ncRNAs with similar patterns in both fluids suggest systemic changes induced by cancer. These findings emphasize the importance of using multiple biological samples for comprehensive biomarker discovery and disease monitoring. Further investigations are needed to understand the biological relationships of ncRNA changes in plasma and sputum.

By analyzing surgically resected tissue samples, Mitchell and colleagues (12) identified seven miRNAs with different expression levels between AA and WA patients with lung cancer. These miRNAs have limited similarity to those identified in plasma and sputum samples. Several reasons could account for these discrepancies: the tissues provide localized information, whereas the plasma and sputum reflect systemic influences. Inherent tumor variability can affect miRNA expression. Tumors may release specific ncRNAs based on their characteristics, with some remaining localized. Furthermore, variations in the laboratory procedures and ncRNA detection methods may yield different results. In addition, genetic and environmental differences within the AA and WA groups can influence ncRNA profiles across studies. In response to this discrepancy, we are currently collecting tissue specimens matched with plasma and sputum samples from various ethnic populations. This will enable us to concurrently profile ncRNA changes and better understand the relationship between molecular aberrations across different specimen types.

The distinctive ncRNA profiles linked to lung cancer in AAs versus WAs may hold promise as biomarkers to address the observed racial disparity in lung cancer. Nonetheless, a large multicenter clinical trial is required to prospectively validate the full utility of biomarkers for early lung cancer detection in different populations.

## Supplementary Material

Supplementary Table 1 Supplementary Table 1. 93 lung cancer-associated ncRNAs tested by ddPCR in this study

Supplementary Table 2 Supplementary Table 2. Among lung cancer patients and cancer-free smokers, 33 ncRNAs showed significantly differential expression in plasma or sputum, irrespective of racial background, as analyzed by the Mann-Whitney U test, with a False Discovery Rate (FDR)-adjusted p-value of less than 0.05.

Supplementary Table 3 Supplementary Table 3. Associations between the ten ncRNAs in the biomarker panels and clinical and demographic data, analyzed using Pearson's correlation coefficients.

Supplementary Table 4 Supplementary Table 4. The diagnostic values of the three individual panels in the validation set.

Supplemental Figure 1 Supplemental Figure 1. Differential expression of eleven ncRNAs in plasma and sputum between AA lung cancer patients and cancer-free smokers.

Supplemental Figure 2 Supplemental Figure 2. Differential expression of 16 ncRNAs in plasma and sputum between WA lung cancer patients and cancer-free smokers.
