# Supplementary material for: Identification of ncRNA Biomarkers in Non–Small Cell Lung Cancer to Address Racial Disparities
Source: Cancer Res Commun. 2024 Dec 27;4(12):3201–8. doi: 10.1158/2767-9764.CRC-24-0262 (PMC11675572; doi:10.1158/2767-9764.CRC-24-0262)
Supplement: Supplementary Table 1 — 93 lung cancer-associated ncRNAs tested by ddPCR in this study [file crc-24-0262_supplementary_table_1_suppst1.docx]

| **Supplemental Table 1.** 93 lung cancer-associated ncRNAs tested by ddPCR in this study |
| --- |
| miR-205-5p, miR-422a, miR-31-5p, miR-944, miR-34a-5p, miR-34b-3p, miR-183-3p, miR-183-5p, miR-147b, miR-221-3p, miR-93-5p, miR-223-3p, miR-145–5p, miR-29c-3p, miR-20a-5p, miR-210-3p, miR-1244-3P, miR-628-3p, miR-448-3p, miR-16-5p, miR-652-5p, let-7a-5p, miR-21-3p, miR-486-5p, miR-155-5p, miR-584-5p, miR-409-5p, miR-326, miR-324-3p, miR-122-5p, miR-103a-3p, miR-30a-5p, miR-1285-3p, miR-1254, miR-574-5p, miR-146b-5p, miR-27a-3p, miR-27b-3p, miR-10a-5p, miR-429, miR-222-3p, miR-125a-5p, miR-125b-5p, miR-124-3p, miR-106a-3p, miR-92a-3p, miR-24a-3p, miR-4753-3p, miR-425-5p, miR-301a-3p, miR-200b-5p, miR-141-3p, miR-204-5p, miR-25-3p, miR-195-5p, miR-152-3p, miR-148a-3p, miR-148b-5p, miR-19a-3p, miR-193a-3p, miR-193b-3p, miR-4251, miR-19b-3p, miR-126-3p, miR-17-5p, miR-375-3p, snoRA3, snoRA14A, snoRA21, snoRA33, snoRA34, snoRA38B, snoRA39, snoRA42, snoRA47, snoRA61, snoRA66, snoRA68, snoaRA71C, snoRA75, snoRA76, snoRA78, snoRA80, snoRA116, snoRD33, snoRD66, snoRD76, and SNHG1, H19, HOTAIR, MEG3, and RMRP. |
