# Supplementary material for: Identification of ncRNA Biomarkers in Non–Small Cell Lung Cancer to Address Racial Disparities
Source: Cancer Res Commun. 2024 Dec 27;4(12):3201–8. doi: 10.1158/2767-9764.CRC-24-0262 (PMC11675572; doi:10.1158/2767-9764.CRC-24-0262)
Supplement: Supplementary Table 2 — Among lung cancer patients and cancer-free smokers, 33 ncRNAs showed significantly differential expression in plasma or sputum, irrespective of racial background, as analyzed by the Mann-Whitney U test, with a False Discovery Rate (FDR)-adjusted p-value of less than 0.05. [file crc-24-0262_supplementary_table_2_suppst2.docx]

| **Supplemental Table 2.** Among lung cancer patients and cancer-free smokers, 33 ncRNAs showed significantly differential expression in plasma or sputum, irrespective of racial background, as analyzed by the Mann-Whitney U test, with a False Discovery Rate (FDR)-adjusted p-value of less than 0.05. | | | | |
| --- | --- | --- | --- | --- |
| ncRNAs | Mean Expression (Cancer Patients) | Mean Expression (Controls) | Mann-Whitney U Statistic | FDR-adjusted p-value |
| Plasma miR-106a-3p | 1.237 | 0.448 | 642 | 0.0011 |
| Plasma miR-145-5p | 2.509 | 1.408 | 747 | 0.0140 |
| Plasma miR-155-5p | 6.003 | 3.537 | 1603 | 0.0091 |
| Plasma miR-19b-3p | 1.541 | 1.216 | 1652 | 0.0410 |
| Plasma miR-20a-5p | 6.052 | 3.565 | 671 | 0.0014 |
| Plasma miR-21-3p | 2.011 | 0.855 | 871 | 0.0487 |
| Plasma miR-326 | 1.876 | 1.234 | 862 | 0.0419 |
| Plasma miR-574-5p | 1.644 | 0.514 | 424 | <0.0001 |
| Plasma miR-628-3p | 2.707 | 1.250 | 402 | <0.0001 |
| Plasma miR-92a-3p | 3.505 | 0.861 | 394 | <0.0001 |
| Plasma miR-210-3p | 5.993 | 4.033 | 644 | 0.0012 |
| Plasma miR-223-3p | 2.606 | 0.765 | 732 | 0.0101 |
| Plasma miR-24a-3p | 5.157 | 3.708 | 643 | 0.0164 |
| Plasma miR-27b-3p | 6.706 | 3.897 | 625 | 0.0107 |
| Plasma miR-29c-3p | 1.664 | 0.836 | 587 | 0.0039 |
| Plasma miR-301a-3p | 2.473 | 1.707 | 676 | 0.0341 |
| Plasma miR-30a-5p | 1.429 | 1.165 | 495 | 0.0002 |
| Plasma miR-486-5p | 2.392 | 0.967 | 479 | 0.0002 |
| Plasma snoRA116 | 1.013 | 0.111 | 656 | 0.0377 |
| Plasma snoRA12 | 4.017 | 1.625 | 3476 | <0.0001 |
| Plasma snoRA47 | 1.892 | 0.857 | 3312 | <0.0001 |
| Plasma snoRA61 | 4.018 | 0.752 | 1467 | 0.0181 |
| Plasma snoRA78 | 3.884 | 0.582 | 1439 | 0.0123 |
| Plasma SNHG1 | 4.023 | 1.407 | 193 | <0.0001 |
| Plasma RMRP | 3.251 | 0.905 | 203 | <0.0001 |
| Sputum miR-126-3p | 1.319 | 0.154 | 707 | <0.0001 |
| Sputum miR-31-5p | 7.379 | 1.467 | 1354 | 0.0312 |
| Sputum miR-486-5p | 13.056 | 6.409 | 1193 | 0.0031 |
| Sputum snoRA42 | 7.867 | 2.452 | 1531 | 0.0370 |
| Sputum snoRA61 | 3.666 | 0.825 | 1511 | 0.0051 |
| Sputum snoRA78 | 4.035 | 0.698 | 1456 | 0.0022 |
| Sputum SNHG1 | 2.013 | 0.682 | 1228 | 0.0120 |
| Sputum H19 | 1.991 | 1.092 | 1538 | 0.0163 |
