# Supplementary material for: Identification of ncRNA Biomarkers in Non–Small Cell Lung Cancer to Address Racial Disparities
Source: Cancer Res Commun. 2024 Dec 27;4(12):3201–8. doi: 10.1158/2767-9764.CRC-24-0262 (PMC11675572; doi:10.1158/2767-9764.CRC-24-0262)
Supplement: Supplementary Table 3 — Associations between the ten ncRNAs in the biomarker panels and clinical and demographic data, analyzed using Pearson's correlation coefficients. [file crc-24-0262_supplementary_table_3_suppst3.docx]

| **Supplemental Table 3**. Associations between the ten ncRNAs in the biomarker panels and clinical and demographic data, analyzed using Pearson's correlation coefficients | | | | | | |
| --- | --- | --- | --- | --- | --- | --- |
| ncRNAs | Age | Sex | Smoking-Pack-Years | Pulmonary Nodule Size | Tumor Stage | Histology |
| Plasma miR-422a | 0.738 | 0.016* | 0.250 | 0.018* | 0.562 | 0.456 |
| Plasma miR-324-3p | 0.995 | 0.001* | 0.588 | 0.060 | 0.752 | 0.264 |
| Plasma miR-147b | 0.472 | 0.414 | 0.877 | 0.091 | 0.008* | 0.819 |
| Plasma miR-205-5p | 0.048* | 0.555 | 0.596 | 0.834 | 0.835 | 0.779 |
| Plasma miR-126-3p | 0.186 | 0.624 | 0.408 | 0.130 | 0.905 | 0.555 |
| Plasma miR-103-3p | 0.571 | 0.191 | 0.983 | 0.055 | 0.934 | 0.561 |
| Sputum miR-34a-5p | 0.092 | 0.676 | 0.925 | 0.521 | 0.226 | 0.307 |
| Plasma miR-21-3p | 0.594 | 0.543 | 0.997 | 0.100 | 0.563 | 0.897 |
| plasma miR-210-3p | 0.200 | 0.115 | 0.614 | 0.542 | 0.313 | 0.688 |
| sputum miR-126-3p | 0.025* | 0.123 | 0.326 | 0.785 | 0.624 | 0.200 |
| * Significance at p < 0.05. | | | | | | |
