# Supplementary material for: Identification of ncRNA Biomarkers in Non–Small Cell Lung Cancer to Address Racial Disparities
Source: Cancer Res Commun. 2024 Dec 27;4(12):3201–8. doi: 10.1158/2767-9764.CRC-24-0262 (PMC11675572; doi:10.1158/2767-9764.CRC-24-0262)
Supplement: Supplementary Table 4 — The diagnostic values of the three individual panels in the validation set. [file crc-24-0262_supplementary_table_4_suppst4.docx]

| **Supplemental Table 4.** The diagnostic values of the three individual panels in the validation set. | | | | | |
| --- | --- | --- | --- | --- | --- |
| Diagnostic performance of the three-biomarker panel in AAs | | Diagnostic performance of the four-biomarker panel in WAs | | Diagnostic performance of the pan-ethnic biomarker panel | |
| Sensitivity, %  (95% CI) | Specificity,  % (95% CI) | Sensitivity, %  (95% CI) | Specificity,  % (95% CI) | Sensitivity,  %(95% CI) | Specificity,  % (95% CI) |
| 85.71%  (67.33% to 95.97%) | 88.89%  (73.94% to 96.89%) | 89.29%  (71.77% to 97.73%) | 86.11%  (70.50% to 95.33%) | 71.43%  (57.79% to 82.70%) | 88.89%  (79.28% to 95.08%) |
