# Supplementary figures and images for: Identification of ncRNA Biomarkers in Non–Small Cell Lung Cancer to Address Racial Disparities
Source: Cancer Res Commun. 2024 Dec 27;4(12):3201–8. doi: 10.1158/2767-9764.CRC-24-0262 (PMC11675572; doi:10.1158/2767-9764.CRC-24-0262)

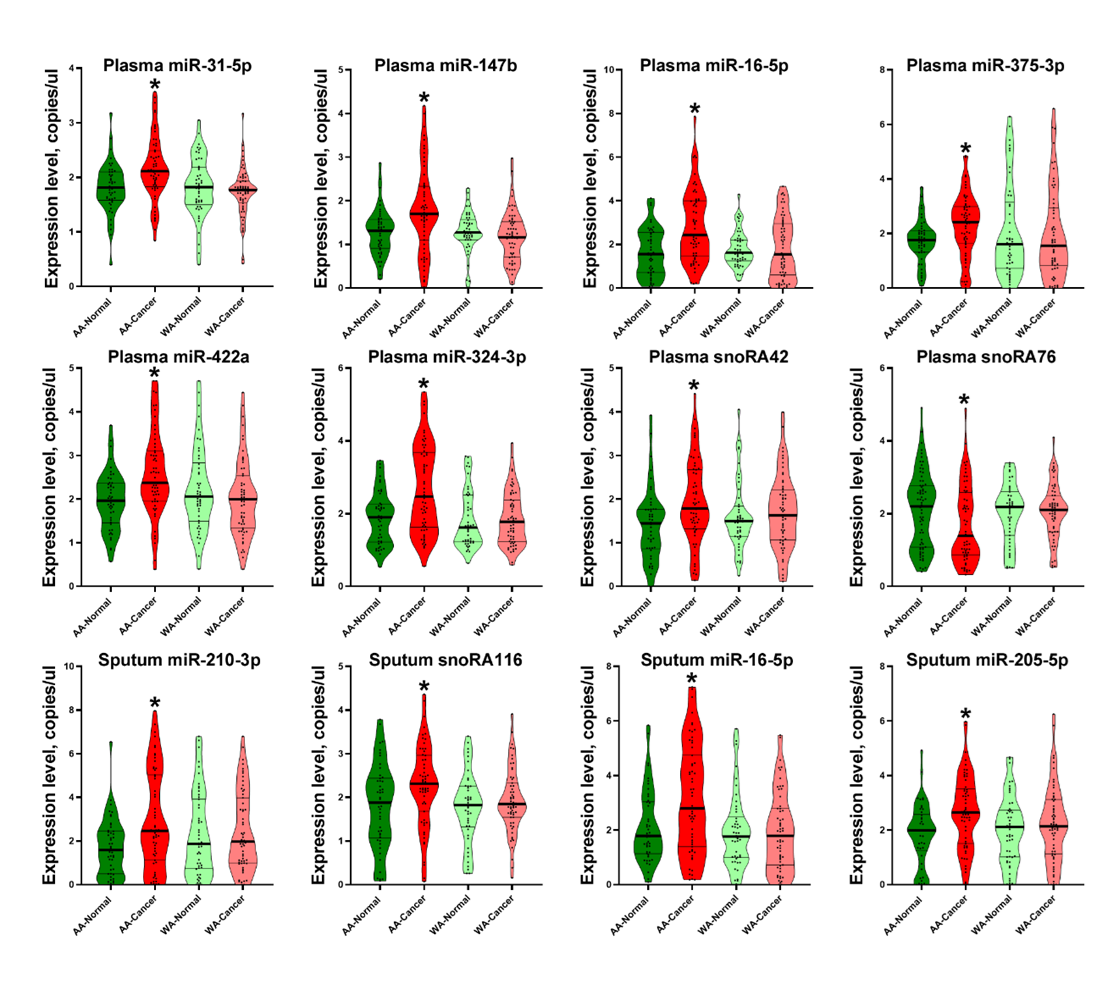

Supplement: Supplemental Figure 1 — Differential expression of eleven ncRNAs in plasma and sputum between AA lung cancer patients and cancer-free smokers. [file crc-24-0262_supplemental_figure_1_suppsf1.png]

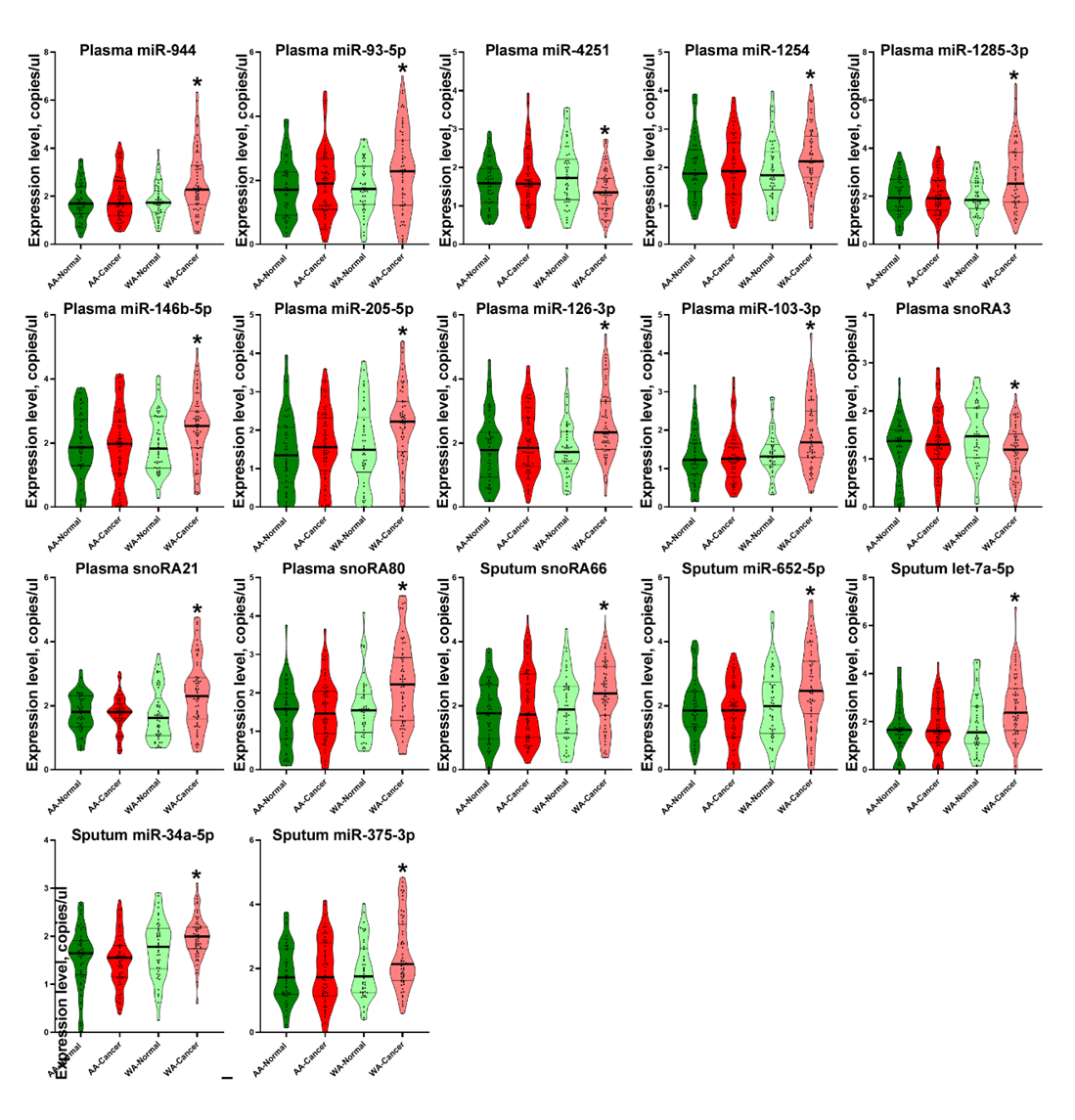

Supplement: Supplemental Figure 2 — Differential expression of 16 ncRNAs in plasma and sputum between WA lung cancer patients and cancer-free smokers. [file crc-24-0262_supplemental_figure_2_suppsf2.png]
